# Reconfiguration and Search of Social Networks

**DOI:** 10.1155/2013/391782

**Published:** 2013-12-18

**Authors:** Lianming Zhang, Aoyuan Peng, Jianping Yu

**Affiliations:** ^1^College of Physics and Information Science, Hunan Normal University, Changsha 410081, China; ^2^Department of Computer Science and Technology, Hunan University of Humanities, Science and Technology, Loudi 417000, China; ^3^College of Mathematics and Computer Science, Hunan Normal University, Changsha 410081, China

## Abstract

Social networks tend to exhibit some topological characteristics different from regular networks and random networks, such as shorter average path length and higher clustering coefficient, and the node degree of the majority of social networks obeys exponential distribution. Based on the topological characteristics of the real social networks, a new network model which suits to portray the structure of social networks was proposed, and the characteristic parameters of the model were calculated. To find out the relationship between two people in the social network, and using the local information of the social network and the parallel mechanism, a hybrid search strategy based on *k*-walker random and a high degree was proposed. Simulation results show that the strategy can significantly reduce the average number of search steps, so as to effectively improve the search speed and efficiency.

## 1. Introduction

The social network is a system which consists of the interpersonal or intergroup relationships. In the system, an individual is abstracted as a node, interpersonal or intergroup social relationships act as the edge between nodes, and they get together to form a social network. Social relations can be many and varied, such as friend relationships between individuals, working relationships between colleagues, marriage relationships between families, and business relationships between companies. The sociologists wish the structural properties of the network can provide a systematic explanation for social phenomena; for example, the average distance of the social network can reflect the speed of information transmission in society. The clustering coefficient reflects the transitivity of social relations, that is, the occurrence possibility of social ties between an individual and his friends' friends. The node degree reflects the frequent interaction among social structures. The node degree distribution reflects the social stratification. Milgram arrived at some inferences as follows through the survey of social relations [1]: The average distance between any two people on the earth is 6, which to some extent reflects the small-world characteristic of interpersonal relationships, but the completion rate of Milgram's experiment is too low. Subsequently, Bacon game about the collaboration network of film actors and Erdös number about the collaboration network of mathematicians confirmed the small-world phenomenon, but they are still too small in size and the statistical properties of the relationship network have low credibility. However, for a fairly long time thereafter, random graph remains the basic theory and the analysis method for the complex structure of the network. But by the end of the 20th century, Watts and Strogatz revealed the small-world characteristic of the complex network [2], that is, large clustering coefficient and short average path length, and Barabási and Albert revealed the scale-free nature of the complex network [3] to establish appropriate models to illustrate the mechanism of these characteristics. Meanwhile, the research team of Watts has done some online experiments on the small-world property of social networks [4]. From then on, people began to consider the overall characteristics of real networks which have large number of nodes and complex structure, such as social networks, information networks, technological networks, and biological networks.

Clearly, to understand the correlation between network structure and network behavior, and to improve the behavior of the network, we need to have a good understanding of the structural features of the real network and on this basis to establish the appropriate network model. Milgram's small-world experiment not only reveals the small-world property of social networks, but also the searchability of social networks. Kleinberg first had shown that in theory [5], a quick search can achieve in the complex networks with small-world characteristic. Since then, Watts et al. made a further study of this problem for social networks [6]. Adamic and Adar had verified some conclusions of the Watts model of social networks based on the small-world experiment of the e-mail network [7]. WS small-world network model explains the small-world property of social networks to a certain extent. However, the small-world property of the network does not necessarily mean that the network can quickly search. Whether a node in the network can find a shorter, or the shortest path between any of other nodes, depends on the network structure information of the node and the search strategy used by the node and the actual structure of the entire network. Therefore, the establishment of the network model and the implementation of efficient search strategy are in favor of finding the shortest chain between two people in the social network, and it has become an important topic in social network analysis.

In this paper, we formulate a deterministic small-world social network model and then put forward a hybrid search strategy based on *k*-walker random and a high degree (KRDS).

## 2. Related Works

### 2.1. Network Model

Nearest-neighbor coupled network (NNCN) model is a sparse regular network model in which each node only connects with its neighbor nodes. Suppose that a NNCN consists of *N* nodes and these *N* nodes are connected into a ring in turn, each node is connected with *K* neighbor nodes and these *K* neighbors are symmetrical to the node as the center. The average path length of the NNCN is *L* ≈ *N*/(2*K*), and the clustering coefficient is *C* = 3(*K* − 2)/(4(*K* − 1)) ≈ 3/4. Obviously, the clustering property of the NNCN is too high, and the average path length is too long, so it does not have the small-world characteristic.

Contrary to the regular network, ER random graph (ERRG) model is a typical representative of random networks [8]. Assume that the total number of the nodes of the random network is *N*, any node connects to other nodes with the same probability *p*. The average path length of the ERRG is *L* ∝ ln⁡(*N*)/ln⁡(〈*k*〉), where 〈*k*〉 = *p*(*N* − 1) ≈ *pN*, and clustering coefficient of the ERRG is *C* = *p* = 〈*k*〉/*N* ≪ 1. Obviously, the ERRG has a smaller average path length, and the clustering coefficient is very small, so it is not a small-world network.

The NNCN and the ERRG cannot reproduce some important features of real networks. After all, most of the real networks are neither completely regular nor completely random. As transition from a completely regular network to a completely random graph, Watts and Strogatz introduced a small-world network (WSSWN) model in 1998 [2]. The WSSWN sets the NNCN as the original model. To an identified edge in the original model, one of its endpoints is fixed, and another endpoint randomly selects a node to reconnect in addition to itself. When the reconnection probability is *p* = 0, the WSSWN is a NNCN. When *p* = 1, the WSSWN is a completely random network. Therefore, the transition from a completely regular network to a completely random network can be controlled by regulating the value of *p*. The average path length of the WSSWN is *L*(*p*) = 2*N*/*Kf*(*NKp*/2) [9], where *f*(*u*) is a universal scaling function, and if *u* ≪ 1, *f*(*u*) = constant and if *u* ≫ 1, *f*(*u*) = ln⁡(*u*)/*u*. The clustering coefficient is *C*(*p*) = 3(*K* − 2)(1−*p*)^3^/(4(*K* − 1)) [10]. When *p* is small (e.g., 0 < *p* ≪ 1), the local properties of the reconnected network and the original regular network are not very different; thus the clustering coefficient of the network will not change much (*C*(*p*) ∝ *C*(0)), but its average path length has declined rapidly (*L*(*p*) ≪ *L*(0)). The network that has a shorter average path length and a higher clustering coefficient is a small-world network.

A common feature of the ERRG and the WSSWN is able to represent the network's connectivity distribution using the Poisson distribution. The distribution has a peak at the average degree 〈*k*〉, and it exponentially decays. This means that when *k* ≫ 〈*k*〉, the *k*-degree nodes are almost nonexistent. Therefore, these networks are also called uniform networks or exponential networks. Barabási and Albert proposed a scale-free network (BASFN) model in 1999 [3]. The node degree of the BASFN has no obvious characteristics, and it is used to analyze the real networks with power-law distribution. The BASFN starts from a network which has *n*_0_ original nodes and then adds a new node each time, where the probability that the new node connects to an old node is ∏_*i*_, and the degree of the node *i* is *k*_*i*_ and the degree of the node *j* is *k*_*j*_. We have the following expression: ∏_*i*_ = *k*_*i*_/∑_*j*_ *k*_*j*_. After *t* steps, the number of the nodes of the result graph is *N* = *t* + *n*_0_. The average path length of the BASFN is *L* ∝ log⁡*N*/loglog*N* [11], and the clustering coefficient is *C* ∝ (ln⁡(*N*))^2^/*N* [12]. Clearly, the BASFN has small average path length, but when *N* is relatively large, the BASFN does not have the clustering characteristic.

In the past 10 years, many varieties of WSSWN and BASFN have been put forward. There are some common models, such as NW small-world model [9], deterministic small-world model [13], multidimensional growth of deterministic small-world network (DMG) model [14], local-world evolving network model [15], and hierarchical network model [16].

### 2.2. Search Strategies

When the source node *s* applies breadth first search (BFS) strategy to search for the target node *t*, *s* first judges whether *t* exists in the neighbor nodes; if any, it is returned to the source node, otherwise, the neighbor nodes pass information to their respective neighbor nodes. Repeat this process until it finds any neighbor node connected with *t*.

When the source node *s* applies depth first search (DFS) strategy to search the target node *t*, *s* first inquires whether *t* exists along a possible branch path, if any, the search is complete, otherwise, it explores as far as possible along each branch before backtracking, and each node can only visit once. Repeat this process until it finds the target node *t*.

When the source node *s* applies random walk search (RWS) strategy to search for the target node *t*, *s* first judges whether its own neighbor nodes have *t*. If any, it stops searching, otherwise, it inspects all the neighboring nodes. Repeat this process until the target node is found [17]. The RWS can be further divided into unrestricted random walk (URW), no-retracing random walk search (NRRW), and self-avoiding random walk search (SARW).

When the source node *s* applies *k*-walker random walk search (*k*-RWS) strategy to search for the target node *t*, *s* will pass *k* copies of queried information to *k* randomly selected neighbors; thus there are *k* visitors to find *t* forward. Each queried information takes its own random walk. Each walker periodically talks with the querying node to decide whether that walker should terminate [18].

When the source node *s* uses high degree search (DS) strategy to search for the target node *t*, *s* searches for the nodes with a maximum degree in its neighbors, if any, it stops searching, otherwise, it keeps searching for the maximum degree one in its all the neighboring nodes [19].

Overall, the BFS strategy can find the shortest path between any two nodes. The shortest path length of the small-world networks is *L* ∝ log⁡*N*, so the BFS strategy in finding the path between the nodes is very effective, and the query is parallel, so the search range will increase exponentially; usually just the inquiries of a few steps will be throughout the network and the search speed is quite fast. However, with the expansion of network size, this approach will produce a large number of querying message flows in the network, resulting in a sharp increase in network traffic and network congestion. The DFS strategy may make the search process along the futile path, resulting in a too long path. It is resolved using the boundary of the depth, and then the path may be the shortest. The number of search steps of the RWS strategy is larger than that of the BFS strategy, and the traffic reduces, but the same time, search speed is greatly reduced. The *k*-RWS strategy makes the search time greatly reduced, and the message traffic generated is much lower than that of the BFS strategy and search performance is better than that of the DFS strategy. The average path length obtained from the DS strategy gets on the same order of magnitude with the average path length obtained from the BFS strategy, and the search speed is fast, but the success rate of search is less than 100%.

## 3. Reconfiguration Method

Real social networks have small-world characteristic, that is, large clustering coefficient and short average path length. Regular network models and random graph models cannot depict the small-world characteristic, and scale-free network models do not have obvious clustering effect. Therefore, the main use of small-world models is to study the structural properties of social networks and to illustrate the generation mechanism of these features. According to the construction methods of the models, small-world networks can be divided into two categories: statistical small-world network models and the deterministic small-world network models. Connection between two nodes in the statistical models is random, and they are not suitable to describe the deterministic relations between the two nodes in social networks. The typical clustering coefficient of the existing deterministic small-world models is about 0.693 [20], and it is less than that of the real social networks, such as the clustering coefficient of the network of film actors which is about 0.78 and that of the networks of company directors which is about 0.88. In this paper, we propose a new social network reconfiguration method and the specific process is as follows.

1. Construct a sphere.
2. For *t* = 0, construct a triangular pyramid, denoted by CUB(0), whose four vertexes are placed in the spherical surface, and each vertex is connected with one of the other three vertexes.
3. For *t* = 1, add a new node on the corresponding sphere of each outside of the triangular pyramid, and add three new edges between the new node and each of three vertexes constructing a corresponding side of the triangular pyramid. It is denoted by CUB(1).
4. After *t* times, create a new graph, denoted by CUB(*t*) in turn.

Through analysis and calculation, we find that, as *t* → *∞*, the node degree distribution function of the model is an exponential distribution, that is, *p* ∝ 3^−log⁡_2_^*k*−1^^ − 3^−log⁡_2_^*k*^^, and the clustering coefficient of the model is *C* ≈ 0.828 and the average path length of the model is *L* ≈ ln⁡(*n*). This shows that the model has small-world property.

When *t* = 8, the network size *N* has reached 13124. Obviously, the construction algorithm of the model has fast convergence and easily constructs large-scale social networks.

## 4. KRDS

In this section, we propose a hybrid search strategy based on *k*-walker random and a high degree (KRDS). The basic ideas of the strategy are as follows: assume that each node in the network knows the information of its neighbor nodes, the source node *s* randomly selects *k* neighbor nodes to transmit information. If there is the target node *t* in these *k* nodes, it will return information. Otherwise, the *k* nodes will, respectively, choose their neighbors with the high degree to pass the information, if the next step still does not find the target node, then use the *k*-walker random to inquire again. Repeat this process until it finds the target node. The pseudocode of the KRDS algorithm as in Algorithm 1.

In Algorithm 1, *k* has a constraint that it will less than or equal to the degree of the current node.

## 5. Simulation and Analysis Results

### 5.1. Search Efficiency

The efficiency of search strategies can be described by the query message traffic in the network and the average number of search steps. The latter reflects the search speed of the strategies, and the former can be measured by the number of traverse nodes from the search process, mainly affecting the network congestion. In this paper, we mainly use the latter to measure the efficiency of the search strategies; that is, the smaller the average number of search steps, the higher the search efficiency. When we put some kind of search strategies for a network with *N* nodes, repeat randomly to select *n* different source nodes. For each selected source node *i*, the sum of the number of the search steps from the source node to all other *N* − 1 nodes based on this search strategy is written by *T*_*i*_ = ∑_*j*=1,*j*≠*i*_^*N*^ *t*_*ij*_. Consequently, the average number of search steps between any two nodes is expressed as *T* = ∑_*i*_^*N*^ *T*_*i*_/[*N*(*N* − 1)].

### 5.2. Simulation Results

In this section, the simulation process is as follows. First, we generate the six network models with different types and sizes: NNCN, ERRG, WSSWN, BASFN, DMG and CUB. Then we assume that each node of the network models only knows its own neighbors and when the source node in the networks finds the target node, and for each model, we apply seven kinds of search strategies: BFS, DFS, DS, URW, NRRW, SARW, and KRDS. Finally, we calculate the average number of search steps of each strategy for each network model.

#### 5.2.1. NNCN

**Figure 1:**
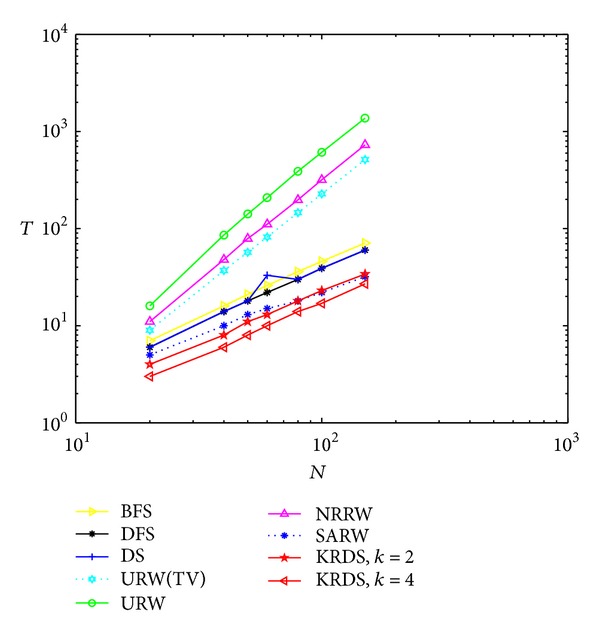
Plot of the average number *T* of the steps versus the network size *N* of the NNCN in the log-log scale.

In the simulation, we generate seven NNCN models with *K* = 4; that is, each node is connected with *K*/2 = 2 neighbors from right and left. The degree of each node is 4. The network size *N* is 20, 40, 50, 60, 80, 100, and 150, respectively. The BFS, the DFS, the DS, the URW, the NRRW, the SARW, and the KRDS (*k* = 2 and *k* = 4) were applied to each network.  Figure 1 shows the impact of the network size *N* on the average number of search steps between any two nodes of the NNCN in the log-log scale.

The average number of search steps from the BFS, the DFS, and the DS strategies is less than that of the NRRW and the SARW strategies, that from the SARW is less than that of the above five strategies, and that from the KRDS strategy is relatively the smallest. For the KRDS strategy, when *k* = 4, the average number of search steps is less than that when *k* = 2. Thus, selecting an appropriate value of *k*, we can greatly reduce the average number of search steps. Pandit and Amritkar derived the theoretical value of the average number of search steps in the application of the URW strategy for searching the NNCN model; that is, *T*_TV_ ≈ (0.17 + 0.14ln⁡(*K*))(*N*/*K*)^2^ [17]. It can be seen that the simulation curves of the average number of search steps of the URW and the NRRW strategies is approximately paralleled with the theoretical curve of the average number of search steps of the URW strategy (see the URW (TV) curve in Figure 1); that is, they approximately obey a power-law relationship: *T* ∝ *N* ^2^. The simulation curves of the average number of search steps from the BFS, the DFS, the DS, the SARW, and the KRDS strategies are almost parallel, but their slope is slightly less than the theoretical value of the URW strategy. Therefore, they approximately obey power-law relationship: *T* ∝ *N* ^*β*^, where 1 < *β* < 2.

#### 5.2.2. ERRG

**Figure 2:**
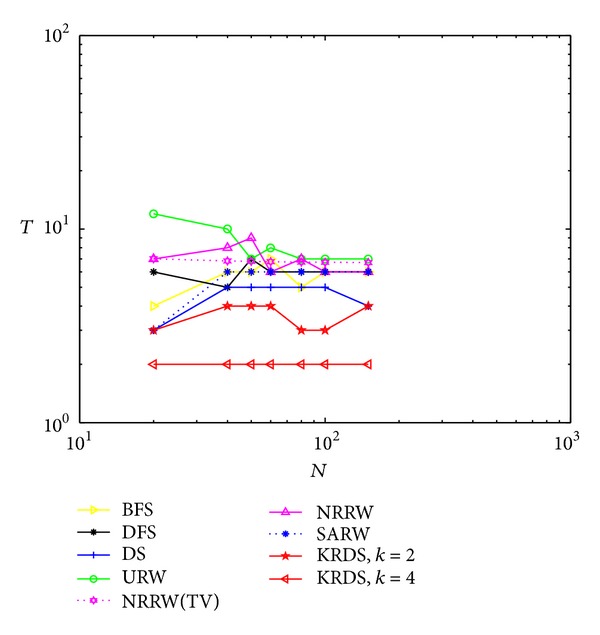
Plot of the average number *T* of the search steps versus the network size *N* of the ERRG with connection probability *p* = 0.15 in the log-log scale.

**Figure 3:**
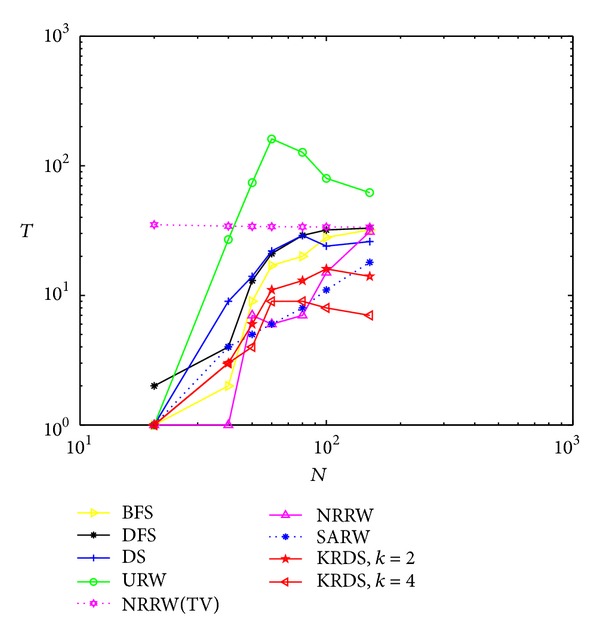
Plot of the average number *T* of the search steps versus the network size *N* of the ERRG with connection probability *p* = 0.03 in the log-log scale.

In the simulation, we generate seven ERRG models with node connection probability *p* = 0.15 and 0.03. The network size *N* of the ERRG is 20, 40, 50, 60, 80, 100, and 150, respectively. The simulation process is similar to the NNCN model. Figures 2 and 3 show the impact of the network size *N* on the average number *T* of search steps of the ERRG with connection probability *p* = 0.15 and *p* = 0.03 in the log-log scale.

Figure 2 shows that the average number of search steps from the URW and the NRRW strategies is the biggest, that of the BFS, the DFS, the DS, and the SARW is less than above, and that of the KRDS is relatively the smallest. The theoretical value of the average number of search steps from the ERRG model based on the NRRW strategy is *T*_TV_ ≈ *N*/(*p*(*N* − 1)). It can be seen when the network size is big, the simulation curves of the average number of search steps obtained by the BFS, the DFS, the URW, the NRRW, and the SARW strategies are approximate equal to the theoretical curve of the average number of search steps from the NRRW strategy (see NRRW (TV) curve in Figure 2). When the network size is small, the average number of search steps obtained by the BFS, the DFS, the URW, the NRRW, and the SARW strategies will be so very different depending on the network size. When the network size reaches a certain value, the average number of search steps has nothing to do with the network size, and that from the DS strategy will appear with the process of the increase, the stability, and the decline with the increase of network size and that from the KRDS (*k* = 2) strategy will appear as fluctuated phenomenon with the increase in the network size. When we select the most appropriate value of *k* (e.g., *k* = 4), the average number of search steps from the KRDS strategy has nothing to do with the network size; it is in good agreement with the theoretical value of the NRRW strategy.

Figure 3 shows that the average number of search steps from the BFS, the DFS, the DS, the NRRW, and the SARW strategies increases with the increase of network size and that from the URW and the KRDS strategies increases first with the increase of network size and then decreases. When the network size reaches a certain value, the simulation value of the average number of search steps from the BFS, the DFS, and the NRRW strategies and the theoretical value of that from the NRRW strategy are in good agreement. When the network size is large, the average number of search steps from the URW strategy is the biggest and that from the KRDS strategy is relatively the smallest. Overall, for different ERRG models for the connection probability *p* = 0.15, the average number of search steps obtained from various strategies is about 5, far from the average number (e.g., 20) of search steps for the connection probability *p* = 0.03. We can know from the critical value of the ERRG model which is *p*_*c*_ ∝ ln⁡(*N*)/*N*, and the seven critical values of different networks above is 0.1498, 0.0922, 0.0782, 0.0682, 0.0548, 0.0461 and 0.0034, respectively. Obviously, the connection probability *p* = 0.15 is greater than the maximum critical value, so the random graph is connected graph, the connection probability *p* = 0.03 is less than the minimum critical value, the random graph is nonconnected graph, and the simulation results are far away from the theoretical values.

**Figure 4:**
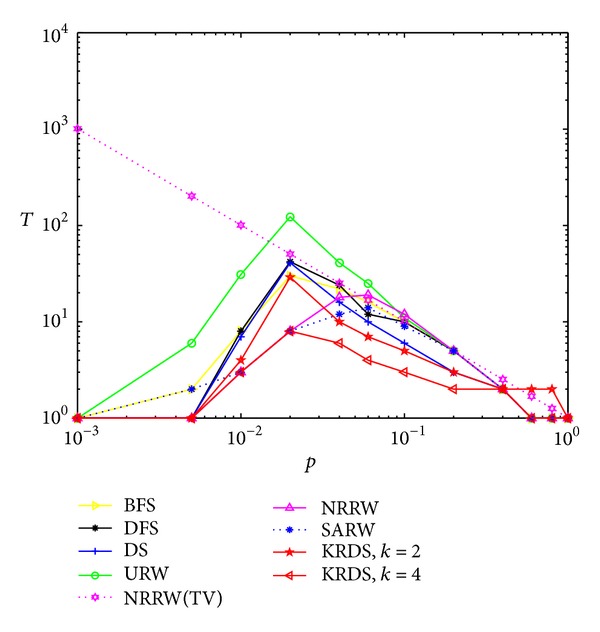
Plot of the average number *T* of search steps versus the connection probability *p* of ERRG in the log-log scale.

In the following section, we consider the average number of search steps for a different node connection probability. In the simulation, we generate twelve ERRG models with the node connection probability *p* = 0.001, 0.005, 0.01, 0.02, 0.04, 0.06, 0.1, 0.2, 0.4, 0.6, 0.8, and 1, respectively, and the network size is all *N* = 100. For each connection probability, using a similar simulation process, the impact of the node connection probability *p* on the average number *T* of search steps of the ERRG models is shown in the log-log scale in Figure 4.

The curves of the average number of search steps from different search strategies have a peak value, and the average number of search steps from the BFS, the DFS, the DS, the URW, and the KRDS strategies to reach the peak corresponding to the connection probability *p* = 0.04 is in good agreement with the theoretical threshold of the ERRG connectivity *p*_*c*_ ∝ ln⁡(*N*)/*N* = 0.0461, while the connection probability of the NRRW and SARW strategies reaching the peak is about 0.06 and there are some deviations with the theoretical value. Obviously, when the ERRG model is the nonconnected graph, that is, the connection probability is less than the critical value, the average number of search steps from each search strategy will increase with the increase of the connection probability. When the ERRG shows the connected graph, the connection probability is greater than the critical value, the average number of search steps from each search strategy will increase with the decrease of connection probability and obey approximate power-law relationship: *T* ∝ *p* ^−*α*^, where *α* > 0. Simulation results of the average number of search steps from the BFS, the DFS, the NRRW, and the SARW strategies and the theoretical value of the NRRW strategy are in good agreement. Overall, the average number of search steps from the URW strategy is the biggest and that from the KRDS (*k* = 4) strategy is relatively the smallest.

#### 5.2.3. WSSWN

**Figure 5:**
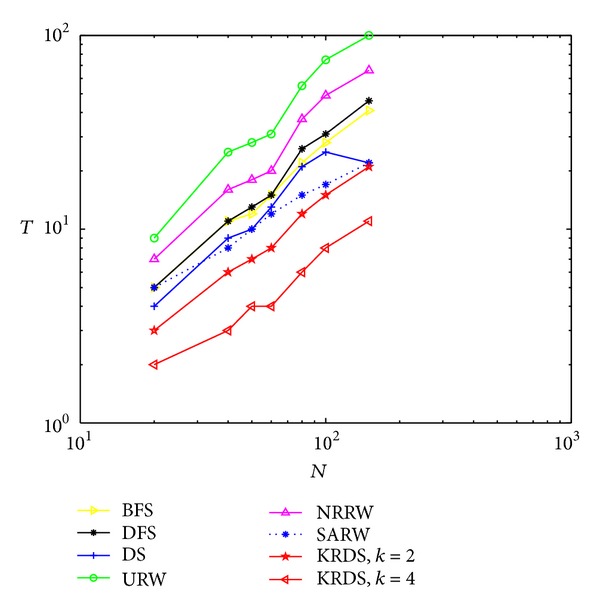
Plot of the average number *T* of search steps versus the network size *N* of the WSSWN with connection probability *p* = 0.3 in the log-log scale.

**Figure 6:**
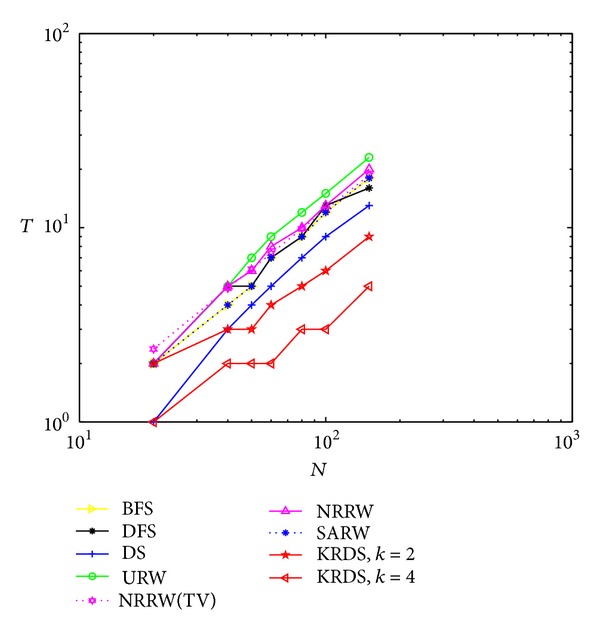
Plot of the average number *T* of search steps versus the network size *N* of the WSSWN with connection probability *p* = 1 in the log-log scale.

We generate seven WSSWNs in the simulation, and the network size is *N* = 20, 40, 50, 60, 80, 100, and 150, respectively; the coefficients of all the NNCNs are *K* = 4 and the reconnection probability of the NNCNs is *p* = 0.3 and 1, respectively. The simulation process is similar to the NNCN and the ERRG. Figures 5 and 6 show the impact of the network size *N* on the average number *T* of search steps of the WSSWN in the log-log scale.

Overall, the relationship between the average number of search steps from a variety of search strategies and the network size of the WSSWN is similar to that between the average number of search steps and the network size of the NNCN. The average number of search steps obtained by each search strategy increases with the increase of network size, and it is similar to the NNCN and approximately obeys the power-law relationship: *T* ∝ *N* ^*β*^. Those obtained by the URW and the NRRW strategies is the biggest and those obtained by the KRDS strategy is relatively the smallest. A WSSWN is the ERRG when the reconnection probability is *p* = 1, so we can apply the NRRW strategy to get the theoretical value of the average number of search steps.  Figure 6 shows that the simulation results of the average number of search steps based on the BFS, the DFS, the URW, the NRRW, and the SARW strategies is closed with the theoretical value of the NRRW strategy.

In the following section, we would consider the average number of search steps obtained by a variety of search strategies from different connection probabilities.

**Figure 7:**
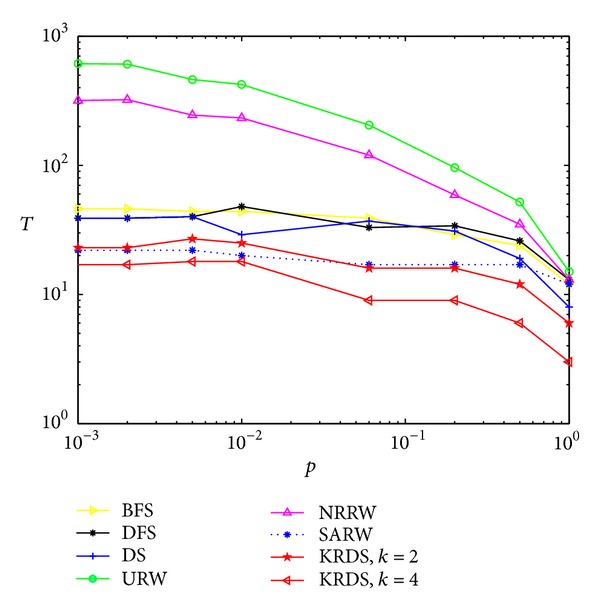
Plot of the average number *T* of search steps versus the connection probability *p* of the WSSWN in the log-log scale.

We generate eight WSSWN models with the reconnection probability *p* = 0.001, 0.002, 0.005, 0.01, 0.06, 0.2, 0.5, and 1, respectively. The network size is *N* = 100, and the coefficients of the all NNCNs are *K* = 4.  Figure 7 shows the impact of the reconnection probability *p* on the average number *T* of search steps of the WSSWN in the log-log scale.

The average number of search steps obtained from various strategies decreases with the increase of *p*, and those from the URW and the NRRW strategies decline the fastest, those from the DS and the KRDS strategies are behind the former, and those from the BFS, the DFS, and the SARW strategies are relatively the smallest. Those from the URW and the NRRW strategies are the biggest, but those from the KRDS strategy are the smallest. When the reconnection probability satisfies 0.01 < *p* < 0.5, the average number *T* of search steps obtained from the URW, the NRRW, and the KRDS strategies and the reconnection probability *p* approximately obey the power-law relationship: *T* ∝ *p* ^−*α*^, where *a* > 0. For the WSSWN model, if *p* = 0, it is a NNCN and if *p* = 1, it is an ERRG. Therefore, if the reconnection probability *p* changes from 0 to 1, the WS small-world network model transfers from a NNCN to an ERRG. The simulation results also reflect such a process. The network is the NNCN with reconnection probability *p* → 0, and the gap between the simulation results of the average number of search steps obtained from the URW, the NRRW, and the SARW strategies and the theoretical value (i.e., 228) of the average number of search steps obtained from the URW strategy is relative small; The network is an ERRG with reconnection probability *p* = 1, and the simulation results of the average number of search steps obtained from the BFS, the DFS, the NRRW and the SARW strategies are closed with the theoretical value (this is 12) of the average number of search steps obtained from the NRRW strategy.

#### 5.2.4. BASFN

**Figure 8:**
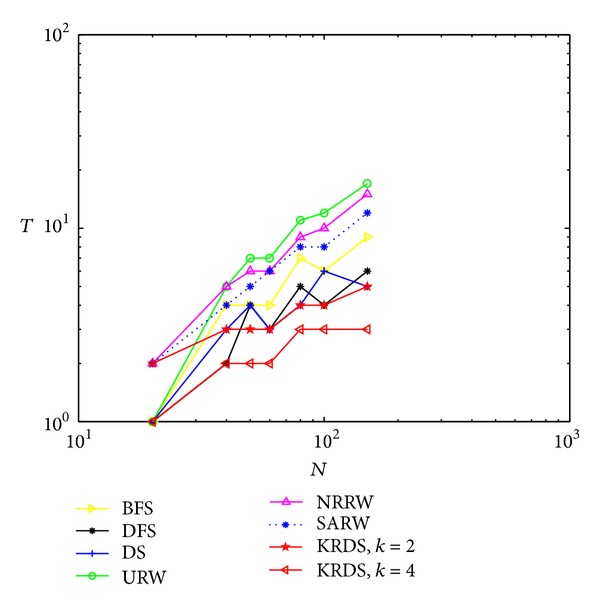
Plot of the average number *T* of search steps versus the network size *N* of the BASFN in the log-log scale.

We generate seven BASFN models in which the number of the original nodes is *m*_0_ = 4, and one new node is introduced and connected to the five nodes each time, and the network size is *N* = 20, 40, 50, 60, 80, 100, and 150, respectively. The simulation process is similar to the former network model.  Figure 8 shows the impact of the network size *N* on the average number *T* of search steps of the BASFN in the log-log scale.

The average number of search steps from three random walk strategies and the KRDS strategy would increase with the increase of network size, but those from the BFS, the DFS, and the DS strategies would increase, reduce and then increase with the increase of network size in the BASFN. When network size is large, the curves obtained from three random walk strategies and the BFS strategy are more or less parallel. The curves from the KRDS strategy using different parameter *k* are roughly parallel for any network size. Thus, they approximately obey power-law relationship: *T* ∝ *N* ^*β*^. Overall, the average number of search steps from three random walk strategies rises the fastest, and the increase rate of the KRDS strategy is relatively the slowest. The average number of search steps from the random walk strategy is the largest and that from the KRDS strategy is relatively the smallest.

#### 5.2.5. DMG

**Figure 9:**
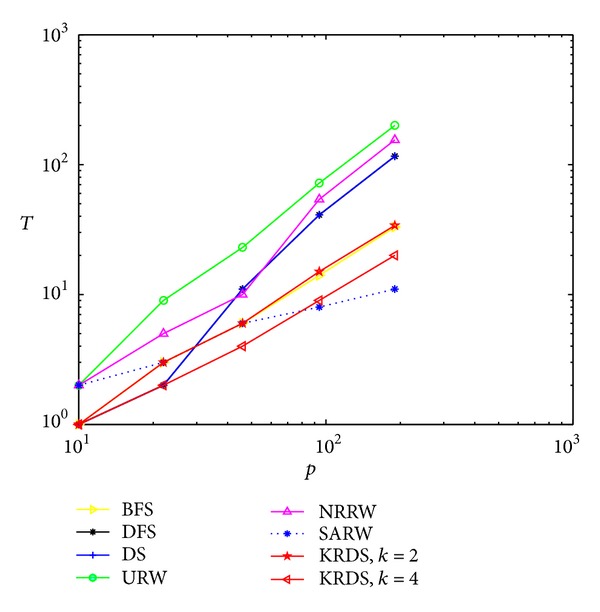
Plot of the average number *T* of search steps versus the network size *N* of the DMG in the log-log scale.

**Figure 10:**
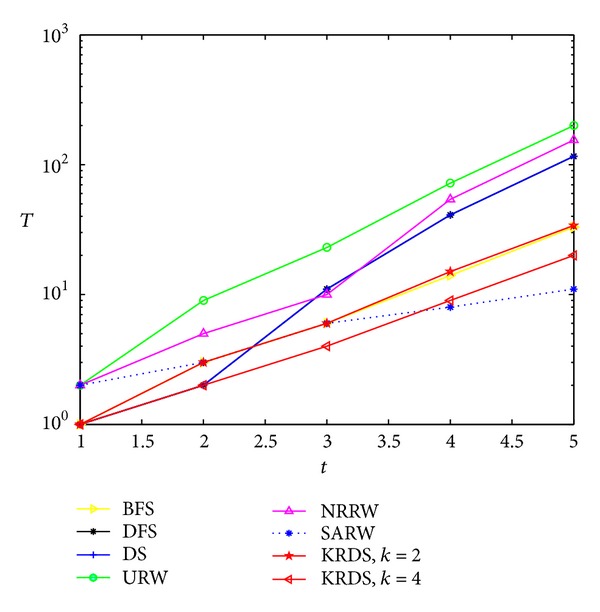
Plot of the average number *T* of search steps versus the number *t* of iterations of the DMG in the semilog scale.

We generate five DMG models, and the network size is *N* = 10, 22, 46, 94, and 190, respectively. Because the relationship of network size and the number of iterations satisfies *N* = 6 × 2^*t*^ − 2, we have *t* = 1, 2, 3, 4, and 5, respectively. Simulation process is similar to the previous four network models.  Figure 9 shows the impact of the network size *N* on the average number *T* of search steps in the DMG model, and Figure 10 shows the impact of the number *t* of iterations on the average number *T* of search steps in the DMG model in the semilogarithmic scale.

The average number of search steps obtained from various strategies would increase with the increase of the number of iterations and the network size and that obtained from the URW strategy is the largest. When the network size is smaller, that obtained from the KRDS (*K* = 4) strategy is relatively the smallest. When the network size is larger, that obtained from the SARW strategy is relatively the smallest. Those obtained from the DFS strategy and the DS strategy are equal. When the network is large, the curves obtained from the DFS, URW, DS, and KRDS strategies are parallel, and they approximately obey power-law relationship: *T* ∝ *N* ^*β*^. In addition, that from the KRDS strategy would decrease with the increase of the parameter *k*, and we can adjust the parameter *k* values to get a smaller average search steps.

#### 5.2.6. CUB

**Figure 11:**
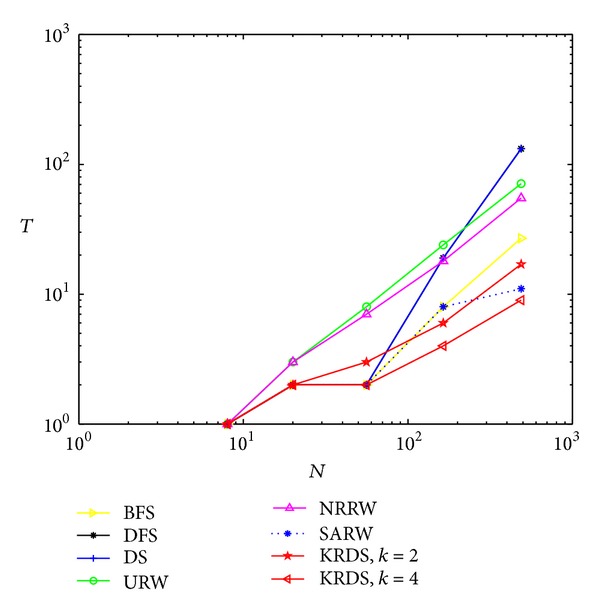
Plot of the average number *T* of search steps versus the network size *N* of the CUB in the log-log scale.

**Figure 12:**
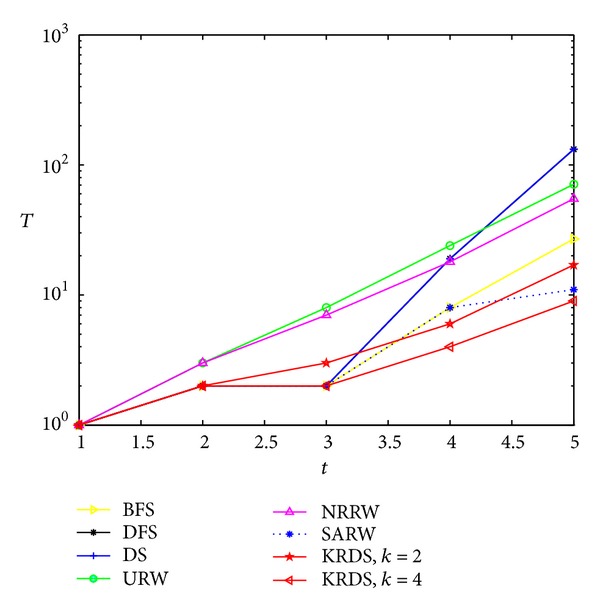
Plot of the average number *T* of search steps versus the number *t* of iterations of the CUB in the semilog scale.

We generate five deterministic small-world network models with spatial structure (CUB), and the network size is *N* = 8, 20, 56, 164, and 488, respectively, and the corresponding number of iterations *t* = 1, 2, 3, 4, and 5. The network size *N* and the number *t* of iterations satisfy the following relationship: *N* = 2 × 3^*t*^ + 2. Simulation process is similar to the previous five network models.  Figure 11 shows the impact of the network size *N* on the average number *T* of search steps in the CUB in the log-log scale, and Figure 12 shows the impact of the number *t* of iterations on the average number *T* of search steps in the CUB in the semilogarithmic scale.

The curve of the average number of search steps obtained from the CUB model is similar to that obtained from the DMG model based on various strategies. However, the former is far less than the latter. When the network size is smaller, the average number of search steps from the URW and NRRW strategies is the largest, but when the network size is large, that from the DS strategy is the largest. That from the KRDS (*K* = 4) strategy is relatively the smallest. When the network size is large, the curves of those from the BFS, the URW, the NRRW, and the KRDS strategies are approximately parallel, and they approximately obey the power-law relationship: *T* ∝ *N* ^*β*^.

### 5.3. Discussion

We have known that the values of the average number of search steps obtained from the same kind of search strategies to different network models are different. Those obtained from different search strategies to the network models with the same type and size are not the same too. In order to understand search capabilities of the different types of networks, such as the social network, and to find the best search strategy of various networks, we have adopted a similar simulation process.

First, we generate the NNCN models, the ERRG models, the WSSWN models, the BASFN models, the DMG models, and the CUB models in succession, and the network size of them is *N* = 164 and 488. The parameters in those models are set as follows: the parameter of the NNCN model is *K* = 4; the connection probability of the ERRG model is *p* = 0.04; the parameters of the WSSWN model are *K* = 4, and *p* = 0.3. The number of the original nodes in the BASFN model is *m*_0_ = 4, and the degree of a new node is *m* = 5. When the network size is 164, the number of iteration steps of the DMG model is set to *t* = 4 and 5 and that of the CUB model is *t* = 4. When the network size is 488, the number of iteration steps of the DMG model is set to *t* = 6 and 7 and that of the CUB model is *t* = 5.

Then, we apply the BFS, DFS, DS, URW, NRRW, SARW, and KRDS (*k* = 2 and 4) strategies to six kinds of the network models to get the average number of search steps. Among them, for the DMG model, we first use iteration steps *t* = 4 and 5 to get the corresponding network size *N* = 94 and 190, respectively. And then, we apply a variety of search strategies to the two networks and can obtain two sets of the average number of search steps. Finally, we estimate the average number of search steps *T*_*N*=164_. The estimation method is as follows: the logarithms of the average number *T* of search steps from various strategies and the network size *N* are approximately linear relationship; that is, log⁡(*T*_*N*=164_)  ≈  (log⁡(*T*_*N*=190_) + log⁡(*T*_*N*=94_))/2, so the value of *T*_*N*=164_ can be calculated. We can calculate the value of *T*_*N*=488_ in the same way.  Table 1 shows that the average number *T* of search steps obtained from a variety of search strategies used in different types of network in which the network size *N* = 164 and 488, respectively.

If the topology structure of the network is different, the average number of search steps is very different; namely, whether the network has the nature of quick search is relative with the network structure. In the above network, the search capability of the NNCN model is the worst, that of the BASFN model is the strongest, and that of the ERRG model is slightly weaker than that of the BASFN model, the search capabilities of small-world networks are stronger than that of the ERRG, but far less than that of the NNCN. In comparison, the BASFN has a bit of high degree nodes, and then there is a bit of long-range connections in the WSSWN, including the ERRG with reconnection probability of 1, but there is a bit of high degree nodes and long-range connections in the CUB, but high degree nodes and long-range connections lead directly to fast search capabilities of the networks. Of course, the proportion of high degree nodes and long-range connections in the network is also very critical, even all are small-world networks and search capabilities are very different. In three small-world networks, the search probability of the CUB is the strongest, that of the DMG is the worst, and that of the WSSWN is between these two. Thus, the small-world characteristic of the network not necessarily means that the network can quickly search.

In addition, for various search strategies, the values of the average number of search steps from the same network are different. That from the URW strategy is the largest, that from the NRRW strategy is a little smaller than that from the URW strategy, and that in the SARW strategy greatly reduces. The values of the average number of search steps from the BFS, DFS, and DS strategies are all a little larger than that of the SARW strategy but are far less than that of the NRRW strategy. In three strategies, that from the DFS strategy is the largest, that from the BFS strategy is the smallest, and that of the DS strategy is between these two. The average number of search steps from the KRDS strategy is the smallest in all strategies. Thus, the search strategy has a significant impact on search efficiency. In comparison, the NRRW strategy avoids the cycle query information between two neighbors in the URW strategy; therefore, the average number of search step has declined; the SARW strategy avoids repeatedly querying each node, and the average number of search steps is relatively small. The BFS strategy is parallel, and the average number of search steps is relatively small, the DFS and the DS strategies need to query individual nodes, so the average number of search steps is slightly larger than that of the BFS strategy. The KRDS strategy is more flexible, and it can get the minimum average number of search steps through adjusting parameter *k* according to the actual network. The KRDS strategy applies the method of *k*-degree parallel query, in every step of search process, and you can obtain *k* times of the node information. At the same time, it avoids querying each node repeatedly. In addition, the average number of search steps of the DMG model from the SARW strategy is the smallest, and that of other various networks from the KRDS strategy is relatively the smallest and the search efficient is the highest.

## 6. Conclusion

In this paper, we proposed a deterministic small-world network model of spatial structure (CUB). The node degree of the network has small-world properties and follows the power-law distribution. Its main characteristics are the ability to portray the social network with a strong clustering coefficient. We proposed the KRDS strategy to find the shortest chain between two people in the social network. The following discoveries were obtained from various search strategies in various network models. (1) The simulation results of the average number of search steps based on the URW strategy of the NNCN model and that based on the NRRW strategy of the ERRG and the WSSWN models are in good agreement with existing theoretical values. (2) The average number *T* of search steps and the network size *N* obtained from various search strategies based on various models obey approximate power-law relationship: *T* ∝ *N* ^*β*^. (3) The average number *T* of search steps and the connection probability *p* obtained from various search strategies based on the ERRG and the WSSWN models obey approximate power-law relationship: *T* ∝ *p* ^−*α*^. (4) The search capability of various networks is different and for small-word network models, the quick search capability of the CUB model proposed in this paper is relatively strongest. (5) The search efficiency of various search strategies is also different, the KRDS strategy proposed in this paper is suitable for most types of network models, and the average number of search steps is the smallest, and in addition, the KRDS strategy only needs to use the local information and would not produce a large amount of network traffic which can quickly get access to a remote connection and effectively improve the efficiency of network search.

## Figures and Tables

**Algorithm 1:**
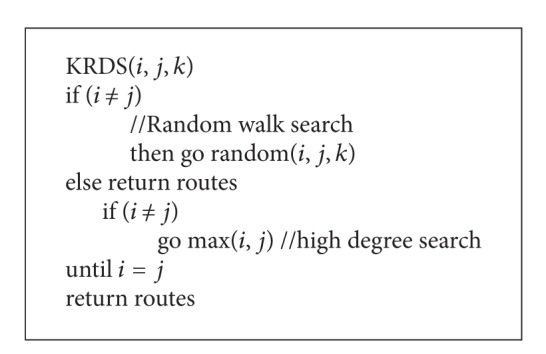


**Table 1:** The number of search steps.

	*N*	BFS	DFS	DS	URW	NRRW	SARW	KRDS (*k* = 2)	KRDS (*k* = 4)
NNCN (*K* = 4)	164	74	56	56	537	410	32	31	21
	488	236	177	177	5075	3838	86	62	64

ERRG (*p* = 0.04)	164	23	23	19	35	29	21	11	6
	488	24	25	17	27	25	25	12	6

WSSWN (*K* = 4, *p* = 0.3)	164	25	30	18	40	32	25	12	6
	488	78	100	54	121	100	71	35	18

BASFN	164	12	8	6	19	17	12	6	3
	488	32	20	16	56	50	35	14	7

DMG	164	21	69	69	119	93	9	23	13
	488	91	425	425	815	646	15	40	41

CUB	164	8	19	19	23	18	8	6	4
	488	27	132	132	71	55	11	17	9
